# Head-to-head comparison of fibroblast activation protein inhibitors (FAPI) radiopharmaceuticals and [^18^F]FDG in gynaecological malignancies: systematic literature review and meta-analysis

**DOI:** 10.1007/s00259-025-07277-0

**Published:** 2025-04-25

**Authors:** Anita Florit, Elizabeth J. de Koster, Serena Sassano, Lejla Alic, Giusi Pisano, Floris H. P. van Velden, Salvatore Annunziata, Irina Primac, Maria Rosaria Ruggiero, Cristina Müller, Evis Sala, Wolfgang P. Fendler, Giovanni Scambia, Lioe-Fee de Geus-Oei, Anna Fagotti, Vittoria Rufini, Angela Collarino

**Affiliations:** 1 Nuclear Medicine Unit, Fondazione Policlinico Universitario A. Gemelli-IRCCS, Largo A. Gemelli, 8, 00168 Rome, Italy; 2 Biomedical Photonic Imaging Group, University of Twente, Enschede, The Netherlands; 3 Department of Surgery, Haaglanden Medical Centre, The Hague, The Netherlands; 4 Section of Nuclear Medicine, Department of Radiology, Leiden University Medical Centre, Leiden, The Netherlands; 5 Section of Nuclear Medicine, Department of Radiological Sciences and Haematology, Università Cattolica del Sacro Cuore, Rome, Italy; 6 Magnetic Detection & Imaging Group, Technical Medical Centre, University of Twente, Enschede, The Netherlands; 7 Radiobiology Unit, Nuclear Medical Applications Institute, Belgian Nuclear Research Centre (SCK CEN), Mol, Belgium; 8 PET/CT Centre, Fondazione Policlinico Universitario A. Gemelli-IRCCS, Rome, Italy; 9 Centre for Radiopharmaceutical Sciences, PSI Center for Life Sciences, Villigen-PSI, Switzerland; 10 Department of Chemistry and Applied Biosciences, ETH Zurich, Zurich, Switzerland; 11 Section of Radiology, University Department of Radiological Sciences and Haematology, Università Cattolica del Sacro Cuore, Rome, Italy; 12 Advanced Radiodiagnostics Centre, Fondazione Policlinico Universitario A. Gemelli-IRCCS, Rome, Italy; 13 Department of Nuclear Medicine, DKTK and NCT University Hospital Essen, Essen, Germany; 14 Gynaecologic Oncology Unit, Department of Woman and Child Health and Public Health, Fondazione Policlinico Universitario A. Gemelli-IRCCS, Rome, Italy; 15 Section of Obstetrics and Gynaecology, University Department of Life Sciences and Public Health, Università Cattolica del Sacro Cuore, Rome, Italy; 16 Department of Radiation Science and Technology, Delft University of Technology, Delft, The Netherlands

**Keywords:** FAPI, [^18^F]FDG, PET/CT, Gynaecological cancers, Systematic review, Meta-analysis

## Abstract

**Purpose:**

This study aims to systematically review and perform a meta-analysis to compare the diagnostic performance of fibroblast activation protein inhibitors (FAPI) radiopharmaceuticals and 2-deoxy-2-[^18^F]fluoro-D-glucose ([^18^F]FDG) in gynaecological cancers.

**Methods:**

A comprehensive search of PubMed/MEDLINE and EMBASE was conducted and updated to October 25, 2024, to identify clinical studies evaluating FAPI and [^18^F]FDG PET/CT or PET/MR in patients with gynaecological cancer. Quality was assessed using the QUADAS-2 tool (Quality Assessment of Diagnostic Accuracy Studies). Per-lesion pooled estimates of sensitivity, specificity, positive predictive value, and negative predictive value were calculated with 95% confidence intervals.

**Results:**

Ten studies were included for qualitative assessment and five studies focusing on ovarian cancer were included in the meta-analysis. The detection rates of primary cervical cancer ranged from 96 to 100% for both radiopharmaceuticals. For the primary tumour in ovarian cancer, the pooled sensitivities of ^68^Ga-FAPI and [^18^F]FDG were 95% and 92%, and the pooled specificities were 81% for both radiopharmaceuticals. Nodal metastases detection was higher with ^68^Ga-FAPI compared with [^18^F]FDG in cervical cancer. Similarly, in ovarian cancer the estimated pooled sensitivities of ^68^Ga-FAPI and [^18^F]FDG were 97% and 88%, and the pooled specificities were 83% and 41%, respectively. At peritoneal metastases analysis in ovarian cancer, the pooled sensitivities of ^68^Ga-FAPI and [^18^F]FDG were 97% and 70%, and the pooled specificities were 93% and 88%, respectively. At the visual assessment of peritoneal cancer scores, such as peritoneal cancer index, ^68^Ga-FAPI detected a greater tumour burden compared with [^18^F]FDG. A comparative analysis of the PET semiquantitative parameters was also performed.

**Conclusion:**

Despite limited literature data, radiopharmaceuticals based on FAPIs are a promising alternative to [^18^F]FDG for imaging gynaecological cancers, in particular for the detection of nodal metastases in cervical and ovarian cancers, as well as for detecting peritoneal metastases in ovarian cancers. Larger prospective studies are needed to confirm these results and promote the inclusion of FAPI radiopharmaceuticals in clinical practice.

**Clinical trial number:**

Not applicable.

**Supplementary Information:**

The online version contains supplementary material available at 10.1007/s00259-025-07277-0.

## Introduction

Gynaecological malignancies comprise a wide range of neoplasms with heterogeneous clinical course and prognosis. Cervical, uterine, and ovarian cancers are the prevalent types, which significantly contribute to high morbidity and mortality rates among gynaecological malignancies [1]. Early diagnosis and accurate staging are essential for implementing the most effective treatment plans. The initial evaluation of patients with gynaecological malignancies typically includes ultrasound and pelvic magnetic resonance (MR) imaging to establish the tumour origin and assess the extent of local disease [2–4]. However, for evaluation of loco-regional and distant involvement, computed tomography (CT) and positron emission tomography (PET)/CT are the preferred imaging modalities [2–4]. To date, the glucose analogue 2-deoxy-2-[^18^F]fluoro-D-glucose ([^18^F]FDG) has been the most widely used radiopharmaceutical for PET/CT in gynaecological cancers [2–5]. [^18^F]FDG uptake in tumour cells is related to upregulation of glucose transporters and hexokinase enzymes, neo-angiogenesis, the number of viable tumour cells, as well as their aggressiveness and proliferative activity [6]. However, the evaluation of [^18^F]FDG uptake can be limited by high physiological background activity in several organs (such as bowel and ureters), variable glucose transporter or hexokinase activity (depending on tumour grading or histology), and low specificity (e.g., increased uptake in acute inflammation) [7, 8]. Additionally, physiological [^18^F]FDG uptake within the endometrial cavity and ovaries during menstrual and ovulatory phases, as well as within benign fibroids or endometriotic cysts, represents a potential pitfall in imaging interpretation [7, 8].

Recently, radiolabelled fibroblast activation protein inhibitors (FAPIs) have emerged as novel radiopharmaceuticals for PET/CT to target tumour microenvironment [9–11]. FAPI selectively binds to fibroblast activation protein (FAP), a type II transmembrane serine protease of the dipeptidyl peptidase-4 family. FAP is primarily overexpressed in cancer-associated fibroblasts, which are integral to the tumour microenvironment and play a key role in cancer aggressiveness and progression [12–14]. In particular, previous studies showed that high FAP expression is a negative prognostic marker for epithelial ovarian cancer and is linked to recurrence after treatment [15]. A series of FAPI ligands (e.g., FAPI-04, FAPI-46, FAPI-74) have already been developed and optimised for stability, uptake, and selectivity, making radiolabelled FAPIs a favourable tool for non-invasive characterization, tumour staging and treatment monitoring [16, 17].

This systematic review and meta-analysis aims at a head-to-head comparison of the diagnostic performance of FAPI radiopharmaceuticals and [^18^F]FDG in gynaecological cancers.

## Materials and methods

### Search strategy and study selection

A systematic literature search was conducted using the PubMed/MEDLINE and Embase databases up to October 25, 2024, in accordance with the Preferred Reporting Items for Systematic Reviews and Meta-analysis (PRISMA) guidelines [18]. The search strategy incorporated synonyms for “gynaecological neoplasm”, “FAPI PET” and “FDG PET” involving the title and abstract, as well as corresponding MeSH terms (Online Resource 1). The full study protocol was prospectively registered (PROSPERO CRD42024593596) and can be viewed online at https://www.crd.york.ac.uk/prospero/display_record.php?ID=CRD42024593596.

### Inclusion and exclusion criteria

Studies were included if they assessed diagnostic performance for detecting primary tumours, loco-regional lymph node metastases, or peritoneal metastases in gynaecological cancers in women aged 18 or older with confirmed disease. Eligible studies were original research involving at least six patients, published in English, and including PET imaging performed with both FAPI radiopharmaceuticals and [^18^F]FDG. Review articles, letters to the editor, editorials, conference abstracts and case reports were excluded. All studies included in the qualitative analysis (systematic review) were also considered in the quantitative meta-analysis if sufficient data were available to assess the diagnostic performances of FAPI and [^18^F]FDG PET/CT or PET/MR. The article selection process involved a comprehensive search of electronic databases, followed by a two-stage screening process. In the first stage, titles and abstracts were independently screened for relevance by two reviewers (A.Fl. and S.S.). In the second stage, full-text articles were assessed for eligibility based on the inclusion and exclusion criteria. Discrepancies between reviewers were resolved through discussion or consultation with a third reviewer (A.C.).

### Data extraction

Data from included studies, capturing publication details (authors, journal, year), study design, funding sources, tumour location, number and subset of patients, were extracted. Information regarding the PET scanner, radiopharmaceuticals administered, time interval between the two scans, reference standards and PET semiquantitative parameters, were recorded. For each included article, relevant data were extracted to construct 2 × 2 contingency tables for per-lesion analysis (primary tumours, loco-regional lymph nodes, and distant metastases). This included the number of true-positives, true-negatives, false-positives and false-negatives. Histopathology and/or follow-up imaging were used as reference standards. The true-positives were defined as lesions showing confirmed pathologic FAPI radiopharmaceutical and/or [^18^F]FDG uptake, while the true-negatives were defined as lesions showing no pathologic FAPI radiopharmaceutical and/or [^18^F]FDG uptake. In case relevant data were missing from studies, the corresponding authors were contacted via email to obtain the necessary parameters.

### Quality assessment

The methodological quality of the included studies was independently evaluated by three authors (A.Fl., E.J.d.K, and S.S.) using the QUADAS- 2 tool for quality assessment of diagnostic accuracy studies [19]. This tool evaluates the risk of bias and the applicability across four key domains: patient selection, index test, reference standard, and flow and timing. The QUADAS- 2 scores for all included articles were tabulated and a summary report was constructed highlighting the strengths and weaknesses of the studies, providing a clear overview of the evidence quality. Any discrepancies in the assessments were resolved through discussion and consensus among the authors, ensuring robust and unbiased evaluations. Based on risk of bias assessments, studies with high risk of bias and applicability concerns across the four key domains were excluded from the meta-analysis.

## Statistical analysis

Studies with adequate data to reconstruct the 2 × 2 contingency table were included in the meta-analysis, focusing on articles evaluating diagnostic performance of FAPI radiopharmaceutical and [^18^F]FDG PET. Meta-analysis was conducted using Stata/MP, version 14.2 (StataCorp LLC, College Station, TX, USA). The metaprop command in Stata/MP and random-effects modelling were used to estimate the pooled sensitivity, specificity, positive predictive value (PPV), negative predictive value (NPV), and their corresponding 95% confidence intervals (CIs). Pooled results are presented in tables and forest plots. Heterogeneity between studies was quantified using the inconsistency index (I^2^). I^2^ ranges from 0 to 100, with values around 0, 25, 50, and 75 representing no, low, moderate, and high heterogeneity among studies, respectively [20].

## Results

### Study selection

**Fig. 1:**
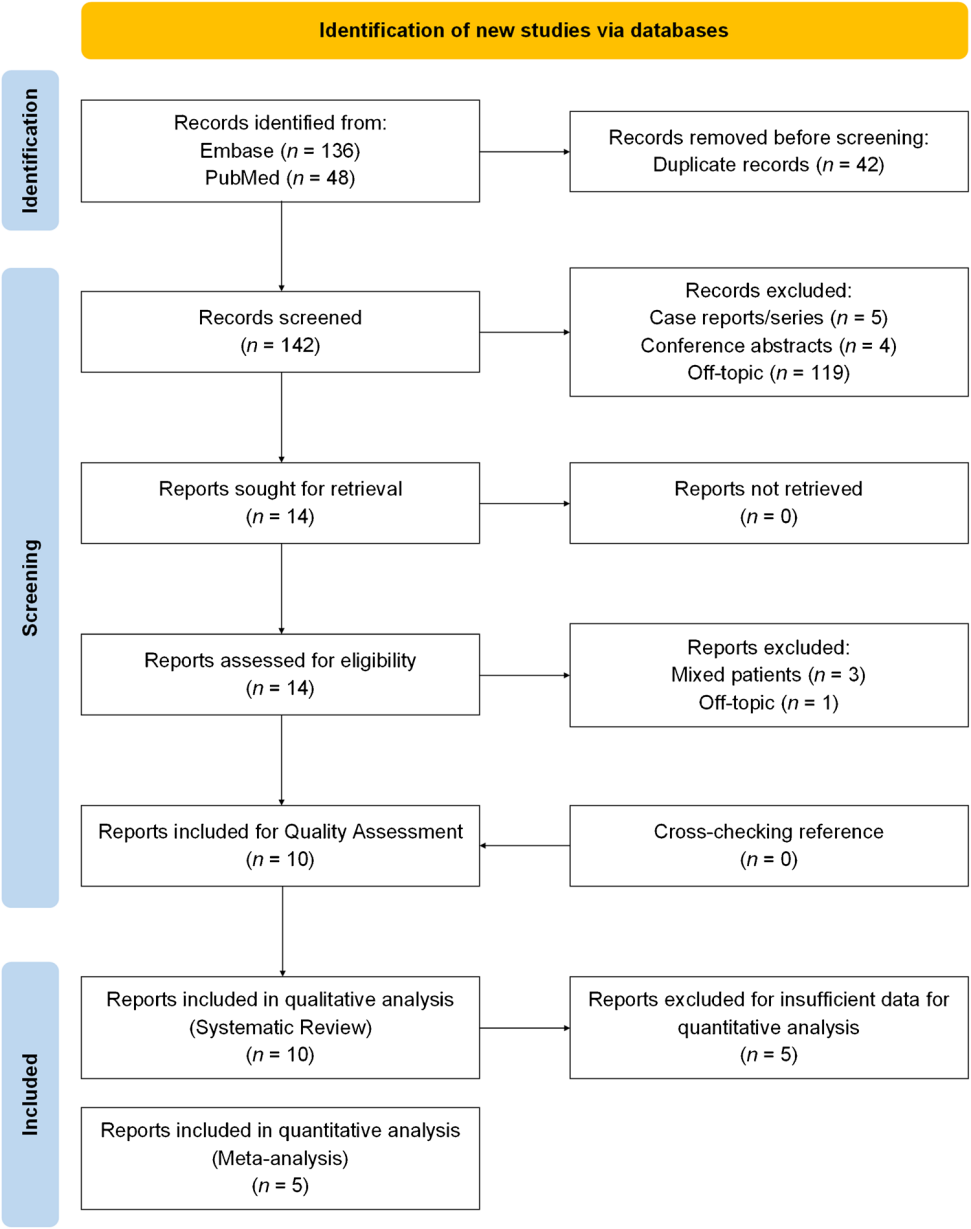
Literature search and study selection

The systematic literature search yielded a total of 184 articles. After removal of duplicates, screening of titles and abstracts, 40 potentially eligible studies were selected. Full-text versions were retrieved. No additional studies were identified during cross-reference checks. Finally, 10 articles were selected for further assessment (Fig. 1) [21–30]. Five out of the 10 studies had adequate data to reconstruct the 2 × 2 contingency table and were therefore included in the meta-analysis.

### Study characteristics

The key characteristics of the included studies are summarised in Table 1 [21–30]. All studies evaluated [^18^F]FDG PET/CT. Seven studies utilised ^68^Ga-FAPI PET/CT [21, 23, 24, 26, 27, 29, 30], while three studies employed ^68^Ga-FAPI PET/MR [22, 25, 28]. One study included patients with a variety of gynaecological cancers [21], four studies focused on cervical and uterine body malignancies [22–25], and five studies investigated ovarian cancer [26–30]. The most commonly used FAPI radiopharmaceutical was [^68^Ga]Ga-FAPI-04 [22, 24–30], with [^68^Ga]Ga-FAPI-46 used in one study [23]. Three different FAP ligands ([^68^Ga]Ga-FAPI-02, −04, −46) were used in one study [21]. In Table 2 the semi-quantitative parameters are listed based on different backgrounds (e.g., liver, mediastinum blood pool, uterus) reported in the included studies.

**Table 1:** Characteristics of the included studies

Authors	Study design	Funding sources	Primary tumour	No. of patients^a^	Patient subset	Intervention	Comparison	Time interval between scans	Reference standard
Dendl et al. [21]	R	Projekt DEAL; Federal Ministry of Education and Research (Germany)	Various gynaecological cancers	10	Staging and restaging	[^68^Ga]Ga-FAPI-02, −04, −46 PET/CT	[^18^F]FDG PET/CT	1–76 days; median: 12.5	Histopathology; F-UP
Zhang et al. [22]	R	National Natural Science Foundation (China)	Cervical and uterine cancers	9	Staging (n = 6) and restaging (n = 3)	[^68^Ga]Ga-FAPI-04 PET/MR	[^18^F]FDG PET/CT	≤ 7 days	Histopathology; F-UP
Wegen et al. [23]	R	SOFIE (for precursors supply)	Cervical cancer	6	Staging	[^68^Ga]Ga-FAPI-46 PET/CT	[^18^F]FDG PET/CT	≤ 6 days	Histopathology
Shu et al. [24]	P	Sichuan Science and Technology Program, Sichuan Provincial Science and Technology Dept., Luzhou Science and Technology Bureau	Cervical cancer	35	Staging	[^68^Ga]Ga-FAPI-04 PET/CT	[^18^F]FDG PET/CT	≤ 7 days	Histopathology; F-UP
Lyu et al. [25]	P	National Natural Science Foundation (China), Natural Science Foundation of Shanghai Science and Technology Commission, Shanghai Municipal Key Clinical Specialty, Shanghai Hospital Development Center Foundation	Cervical cancer	25	Staging	[^68^Ga]Ga-FAPI-04 PET/MR	[^18^F]FDG PET/CT	≤ 14 days	Histopathology; F-UP
Zheng et al. [26]	R	Nuclear Medicine and Molecular Imaging Key Laboratory of Sichuan Province	Ovarian cancer	21	Staging (n = 11) and restaging (n = 10)	[^68^Ga]Ga-FAPI-04 PET/CT	[^18^F]FDG PET/CT	≤ 7 days	Histopathology; F-UP
Liu S. et al. [27]	P	National Key Research and Development Program (China); Shanghai Anticancer Association Program; Shanghai Hospital Development Center; Shanghai Pudong Scientific and Technological development Projects	Ovarian cancer	29	Restaging	[^68^Ga]Ga-FAPI-04 PET/CT	[^18^F]FDG PET/CT	≤ 7 days	Histopathology; F-UP
Xi et al. [28]	P	National Natural Science Foundation (China); Natural Science Foundation of Shanghai Science and Technology Commission; Shanghai Municipal Key Clinical Specialty	Ovarian cancer	30	Staging	[^68^Ga]Ga-FAPI-04 PET/MR	[^18^F]FDG PET/CT	≤ 5 days	Histopathology
Chen et al. [29]	P	Improvement Project for Theranostic Ability on Difficulty Miscellaneous Disease (tumor); National Natural Science Foundation (China)	Ovarian cancer	49	Staging (n = 28) and restaging (n = 21)	[^68^Ga]Ga-FAPI-04 PET/CT	[^18^F]FDG PET/CT	≤ 7 days	Histopathology; F-UP
Liu Y. et al. [30]	P	Foundation of Hebei Provincial Dept. of Education Degree Office Graduate Innovation Funding Project for Higher Education Institutions; Hebei Provincial Medical Applicable Technology Tracking Project; Health Commission Foundation of Hebei Province	Ovarian cancer	79	Staging (n = 6) and restaging (n = 73)	[^68^Ga]Ga-FAPI-04 PET/CT	[^18^F]FDG PET/CT	≤ 7 days	Histopathology; F-UP

^a^ Patients included in the comparative analysis between ^68^Ga-FAPI and [^18^F]FDG PET imaging. F-UP: clinical-imaging follow-up. FAPI: fibroblast activation protein inhibitors. FDG: 2-deoxy- 2-fluoro-D-glucose. P: Prospective study. PET/CT: positron emission tomography/computed tomography. PET/MR: PET/Magnetic resonance imaging. R: Retrospective study

**Table 2:** Semiquantitative parameters described in the included studies

Authors	Parameter(s)	Tumour/background ratio	Significant parameters
Dendl et al. [21]	SUV_max_ SUV_mean_ TBR (SUV_max_/SUV_mean_)	Lymph nodes/fat tissue; bone/bone spongiosa; liver/liver parenchyma; lung/lung parenchyma; tumour/blood pool; tumour/muscle; tumour/fat tissue	TBR-distant metastases
Zhang et al. [22]	SUV_max_	N/A	N/A
Wegen et al. [23]	CR_max_ CR_peak_ TBR_max_ (CR_max_/CR_mean_) TBR_peak_ (CR_peak_/CR_mean_)	Primary tumour/liver; primary tumour/blood pool; metastasis/liver; metastasis/blood pool	TBR_max_-tumour/liver; TBR_peak_-tumour/liver; TBR_max_-tumour/blood pool
Shu et al. [24]	SUV_max_	N/A	None
Lyu et al. [25]	SUV_max_ TBR (SUV_max_/SUV_max_)	Tumour/uterus; tumour/pelvic bowel; tumour/liver blood pool; tumour/mediastinum	TBR-tumour/uterus; TBR-tumour/pelvic bowel; TBR-tumour/liver; TBR-tumour/mediastinum
Zheng et al. [26]	SUV_max_ TBR (SUV_max_/SUV_mean_)	Not specified	TBR-tumour; TBR-lymph nodes; TBR-peritoneal metastases
Liu S. et al. [27]	SUV_max_ TBR (SUV_max_/SUV_max_)	Primary tumour/liver; lymph nodes/liver; distant metastases/liver	TBR-lymph nodes/liver; TBR-distant metastases/liver
Xi et al. [28]	SUV_max_ TBR (SUV_max_/SUV_max_)	Tumour/mediastinum; tumour/liver; uterine metastases/uterus	TBR-tumour/liver; TBR-peritoneal metastases/mediastinum; TBR-peritoneal metastases/liver; TBR-peridiaphragmatic metastases/mediastinum; TBR-uterine metastases/uterus
Chen et al. [29]	SUV_max_ SUV_mean_ FTV/MTV TLF/TLG TBR (SUV_max_/SUV_mean_)	Tumour/liver	TBR-primary tumour/liver; TBR-lymph nodes/liver; TBR-distant metastases/liver FTV/MTV; TLF/TLG
Liu Y. et al. [30]	SUV_max_ TBR (SUV_max_/SUV_mean_)	Not specified	TBR-peritoneal metastases

CR: count rate. FTV: FAP-expressing tumour volume. MTV: metabolic tumour volume. N/A: not available. SUV: standardised uptake value. TBR: tumour-to-background ratio. TLF: total lesion FAP expression. TLG: total lesion glycolysis

### Quality assessment

Table 3 summarises the results of the quality assessment. Regarding risk of bias, most of the studies were assessed as unclear risk in the domain of patient selection because the method of patients’ enrolment was not reported (i.e., consecutive or random). As for the reference standard domain, most studies were rated as unclear because they did not report whether the interpretation of the reference standard results was blinded to the index test results. Regarding applicability concerns, most of the studies generally raised low concerns across the evaluated domains.

**Table 3:** QUADAS-2 methodological quality assessment

		Risk of Bias					Applicability Concerns		
Study		Patient Selection	Index Test	Reference Standard	Flow and Timing		Patient Selection	Index Test	Reference Standard
Dendl et al. [21]		?	?	?	☹		☺	☺	☺
Zhang et al. [22]		?	?	?	?		☺	☺	☺
Wegen et al. [23]		☺	?	?	☺		☺	☺	☺
Shu et al. [24]		?	☺	?	?		☺	☺	☺
Lyu et al. [25]		?	?	?	☺		☺	☺	☺
Zheng et al. [26]		?	☺	☹	☹		☺	☺	☺
Liu S. et al. [27]		?	☺	?	?		☺	☺	☺
Xi et al. [28]		?	☺	?	☺		☹	☺	☺
Chen et al. [29]		?	?	?	?		☺	☺	☺
Liu Y. al. [30]		?	?	☹	?		☺	☺	☺

☺: low risk;?: unclear risk; ☹: high risk

### Comparison of ^68^Ga-FAPI and [^18^F]FDG in detecting primary tumour

[^68^Ga]Ga-FAPI-04 and [^18^F]FDG uptake of the primary tumour was high and diffuse in two patients with uterine cancers (^68^Ga-FAPI maximum standardised uptake value (SUV_max_) 13.4 and 24.2 vs [^18^F]FDG-SUV_max_ 21.7 and 9.3, respectively) [22].

The detection rate of the primary tumour ranged from 96 to 100% for both [^68^Ga]Ga-FAPI-04 and [^18^F]FDG in 60 cervical cancer patients [24, 25]. No uptake of [^68^Ga]Ga-FAPI-04 or [^18^F]FDG was found in one patient with clear cell carcinoma [25]. At semi-quantitative analysis of PET parameters, tumour-to-liver ratios of ^68^Ga-FAPI were significantly higher than [^18^F]FDG [23, 25]. Conversely, the ratio between tumour and uterine myometrium SUVs was significantly lower for [^68^Ga]Ga-FAPI-04, compared with [^18^F]FDG (1.62 ± 1.28 vs 4.07 ± 2.70) [25].

Per-lesion analysis of the primary tumour was carried out in four studies, including 66 patients with suspected or biopsy-proven ovarian cancer (109 lesions). The pooled sensitivities of [^68^Ga]Ga-FAPI-04 and [^18^F]FDG were 95% and 92%, respectively; the pooled specificities were 81% for both radiopharmaceuticals; the pooled PPVs were 97% for both radiopharmaceuticals; the pooled NPVs were 69% and 46%, respectively (Table 4; Online Resource 2) [26, 28–30].

**Table 4:** Data for per-lesion qualitative analysis of the primary tumour in ovarian cancer

	Sensitivity (95% CI)		Specificity (95% CI)		PPV (95% CI)		NPV (95% CI)	
	^68^Ga-FAPI	[^18^F]FDG	^68^Ga-FAPI	[^18^F]FDG	^**6**8^Ga-FAPI	[^18^F]FDG	^68^Ga-FAPI	[^18^F]FDG
Zheng et al. [26]	1.00 (0.78, 1.00)	0.79 (0.52, 0.92)	-	-	1.00 (0.78, 1.00)	1.00 (0.74, 1.00)	-	0.00 (0.00, 0.56)
Xi et al. [28]	0.89 (0.75, 0.96)	0.95 (0.82, 0.99)	0.78 (0.45, 0.94)	0.78 (0.45, 0.94)	0.94 (0.81, 0.98)	0.95 (0.82, 0.99)	0.64 (0.35, 0.85)	0.78 (0.45, 0.94)
Chen et al. [29]	0.94 (0.80, 0.98)	0.91 (0.76, 0.97)	0.80 (0.38, 0.96)	0.80 (0.38, 0.96)	0.97 (0.84, 0.99)	0.97 (0.84, 0.99)	0.67 (0.30, 0.90)	0.57 (0.25, 0.84)
Liu Y. et al. [30]	1.00 (0.72, 1.00)	0.90 (0.60, 0.98)	1.00 (0.21, 1.00)	1.00 (0.21, 1.00)	1.00 (0.72, 1.00)	1.00 (0.70, 1.00)	1.00 (0.21, 1.00)	0.50 (0.09, 0.91)
**Pooled** **[I**^**2**^**, *p*-value]**	0.95 (0.91, 1.00) [4.00, 0.37]	0.92 (0.87, 0.97) [0.00, 0.56]	0.81 (0.61, 1.01) [0.00, 0.80]	0.81 (0.61, 1.01) [0.00, 0.80]	0.97 (0.93, 1.01) [0.00, 0.77]	0.97 (0.93, 1.01) [0.00, 0.83]	0.69 (0.48, 0.90) [0.00, 0.56]	0.46 (0.07, 0.86) [77.41, 0.00]

### Comparison of ^68^Ga-FAPI and [^18^F]FDG in detecting nodal metastases

The ^68^Ga-FAPI detection rate of metastatic lymph nodes was higher than [^18^F]FDG in three studies including 48 patients with cervical cancer [22–24]. In detail, Zhang et al. detected 37 additional nodal metastases in two patients using [^68^Ga]Ga-FAPI-04 [22]. Wegen et al. and Shu et al. reported two and one additional metastatic lymph nodes identified by ^68^Ga-FAPI, respectively [23, 24]. Moreover, in one study [^18^F]FDG identified 12 false positive pelvic and distant nodes in eight patients, in another study 12 false positive nodes (two of which para-aortic) in nine patients, none of which were detected by [^68^Ga]Ga-FAPI-04 [24, 25]. As reported by Lyu et al., the specificity of [^68^Ga]Ga-FAPI-04 in detecting metastatic nodes was significantly higher than [^18^F]FDG for 25 patients (100% vs 59.1% per-patient, respectively) [25]. At semi-quantitative analysis of PET parameters, the ratio between nodal metastases and liver count rates of [^68^Ga]Ga-FAPI-46 was higher, compared with [^18^F]FDG [14.55 (median; range, 12.71–23.10) vs 1.39 (median; range, 1.30–3.41)] [23].

**Fig. 2:**
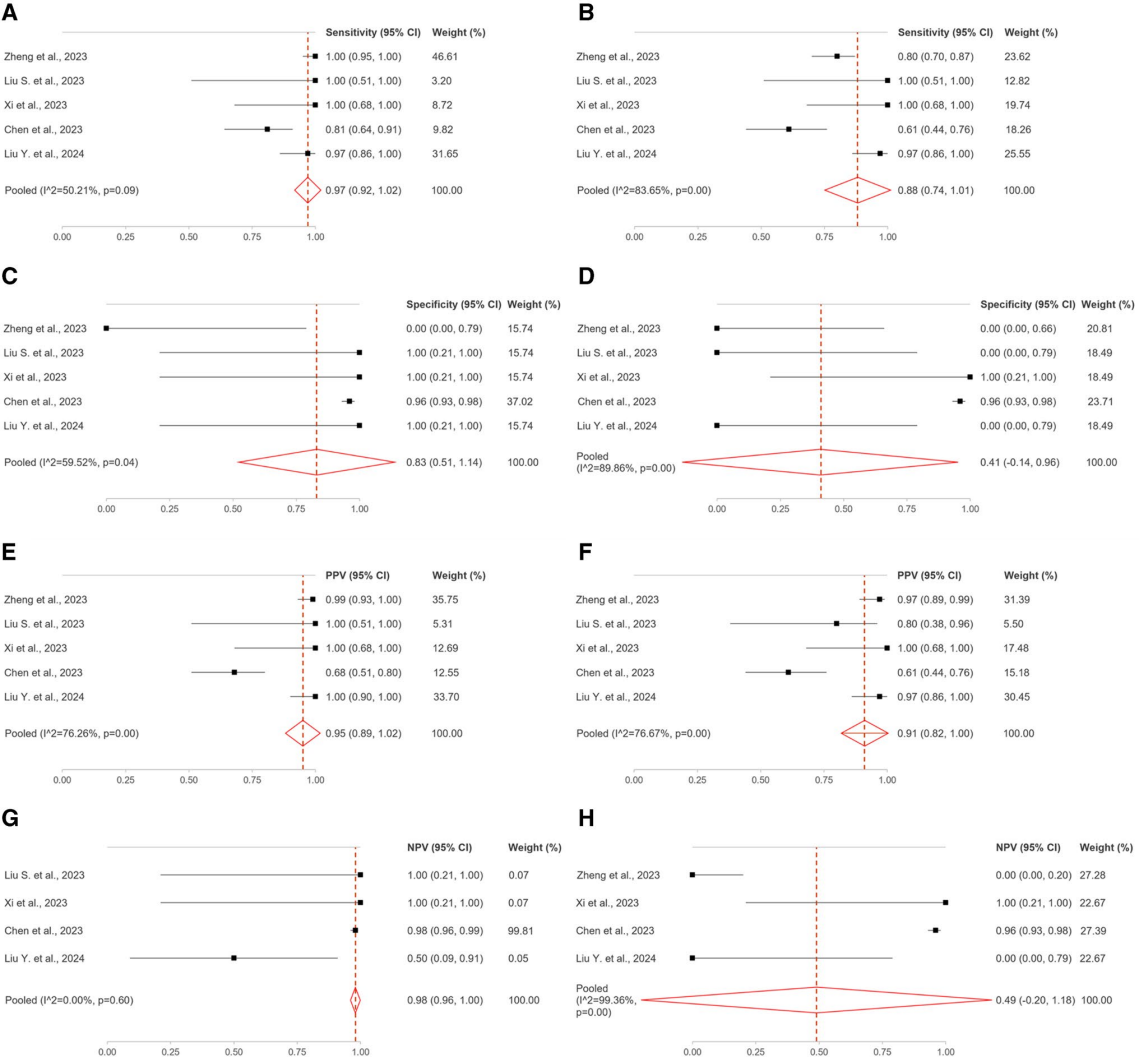
Per-lesion analysis of the nodal metastases in ovarian cancer. (**A**) ^68^Ga-FAPI sensitivity; (**B**) [^18^F]FDG sensitivity; (**C**) ^68^Ga-FAPI specificity; (**D**) [^18^F]FDG specificity; (**E**) ^68^Ga-FAPI positive predictive value (PPV); (**F**) [^18^F]FDG PPV; (**G**) ^68^Ga-FAPI negative predictive value (NPV); (H) [^18^F]FDG NPV

Per-lesion analysis of the nodal metastases was carried out in five studies including 104 ovarian cancer patients (464 lesions). The pooled sensitivities of [^68^Ga]Ga-FAPI-04 and [^18^F]FDG were 97% and 88%, respectively; the pooled specificities were 83% and 41%, respectively; the pooled PPVs were 99% and 91%, respectively; the pooled NPVs were 86% and 49%, respectively (Table 5; Fig. 2) [26–30]. At semi-quantitative analysis of PET parameters, the median SUV_max_ and tumour-to-background ratio (TBR) of nodal metastases were significantly higher with [^68^Ga]Ga-FAPI-04 compared with those of [^18^F]FDG (SUV_max_: 7.0 vs 4.4; TBR 7.0 vs 2.2) [26].

**Table 5:** Data for per-lesion qualitative analysis of the nodal metastases in ovarian cancer

	Sensitivity (95% CI)		Specificity (95% CI)		PPV (95% CI)		NPV (95% CI)	
	^68^Ga-FAPI	[^18^F]FDG	^68^Ga-FAPI	[^18^F]FDG	^68^Ga-FAPI	[^18^F]FDG	^68^Ga-FAPI	[^18^F]FDG
Zheng et al. [26]	1.00 (0.95, 1.00)	0.80 (0.70, 0.87)	0.00 (0.00, 0.79)	0.00 (0.00, 0.66)	0.99 (0.93, 1.00)	0.97 (0.89, 0.99)	-	0.00 (0.00, 0.20)
Liu S. et al. [27]	1.00 (0.51, 1.00)	1.00 (0.51, 1.00)	1.00 (0.21, 1.00)	0.00 (0.00, 0.79)	1.00 (0.51, 1.00)	0.80 (0.38, 0.96)	1.00 (0.21, 1.00)	-
Xi et al. [28]	1.00 (0.68, 1.00)	1.00 (0.68, 1.00)	1.00 (0.21, 1.00)	1.00 (0.21, 1.00)	1.00 (0.68, 1.00)	1.00 (0.68, 1.00)	1.00 (0.21, 1.00)	1.00 (0.21, 1.00)
Chen et al. [29]	0.81 (0.64, 0.91)	0.61 (0.44, 0.76)	0.96 (0.93, 0.98)	0.96 (0.93, 0.98)	0.68 (0.51, 0.80)	0.61 (0.44, 0.76)	0.98 (0.96, 0.99)	0.96 (0.93, 0.98)
Liu Y. et al. [30]	0.97 (0.86, 1.00)	0.97 (0.86, 1.00)	1.00 (0.21, 1.00)	0.00 (0.00, 0.79)	1.00 (0.90, 1.00)	0.97 (0.86, 1.00)	0.50 (0.09, 0.91)	0.00 (0.00, 0.79)
**Pooled** **[I**^**2**^**, *p*-value]**	0.97 (0.92, 1.02) [50.21, 0.09]	0.88 (0.74, 1.01) [83.65, 0.00]	0.83 (0.51, 1.14) [59.52, 0.04]	0.41 (−0.14, 0.96) [89.86, 0.00]	0.95 (0.89, 1.02) [76.26, 0.00]	0.91 (0.82, 1.00) [76.67, 0.00]	0.98 (0.96, 1.00) [0.00, 0.60]	0.49 (−0.20, 1.18) [99.36, 0.00]

### Comparison of ^68^Ga-FAPI and [^18^F]FDG in detecting distant metastases

[^68^Ga]Ga-FAPI-04 uptake of uterine metastases was lower than that of [^18^F]FDG in one cervical cancer patient ([^68^Ga]Ga-FAPI-SUV_max_ 3.6 vs [^18^F]FDG-SUV_max_ 7.5) [22].

**Fig. 3:**
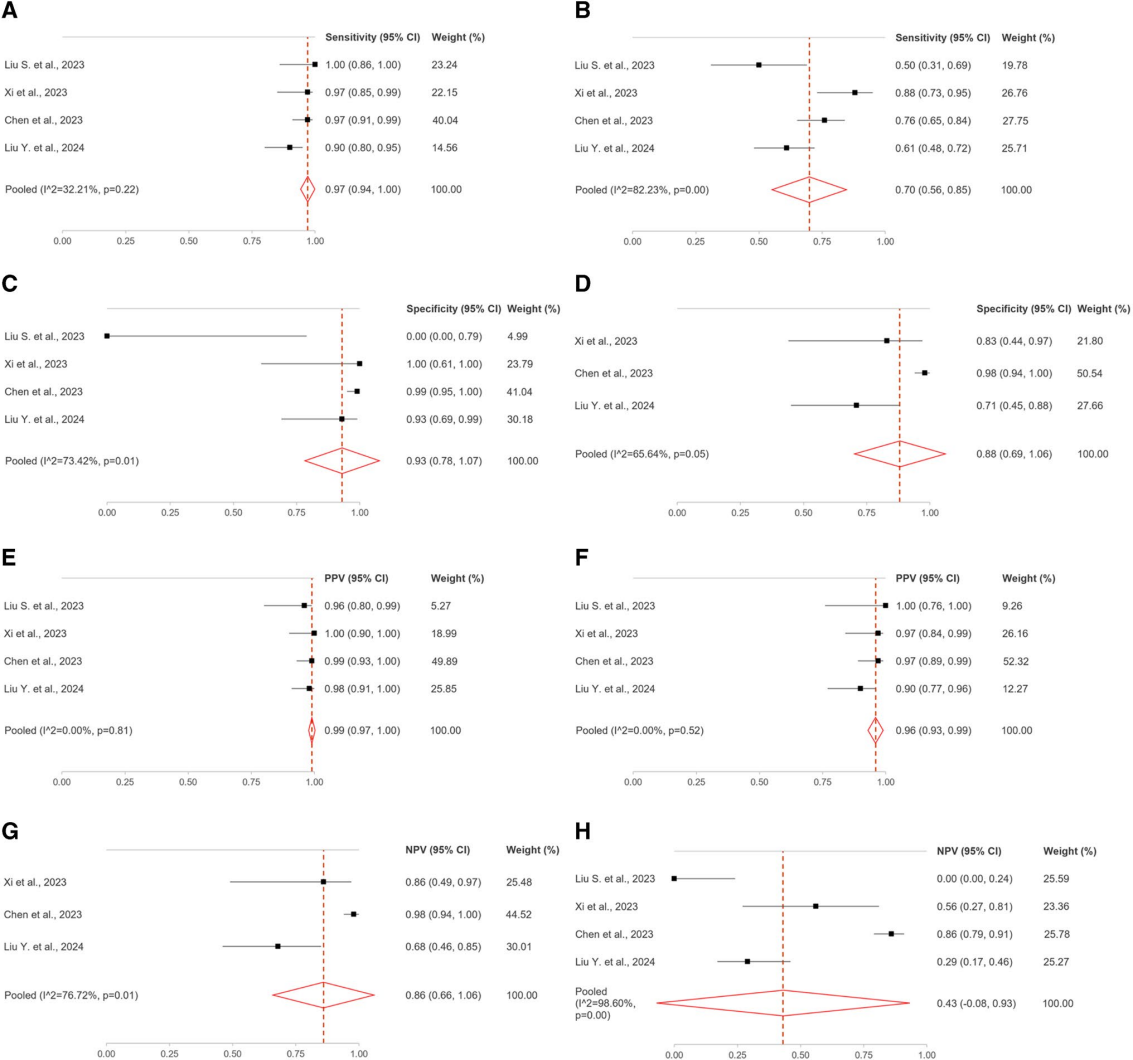
Per-lesion analysis of the peritoneal metastases in ovarian cancer. (**A**) ^68^Ga-FAPI sensitivity; (**B**) [^18^F]FDG sensitivity; (**C**) ^68^Ga-FAPI specificity; (**D**) [^18^F]FDG specificity; (**E**) ^68^Ga-FAPI positive predictive value (PPV); (**F**) [^18^F]FDG PPV; (**G**) ^68^Ga-FAPI negative predictive value (NPV); (H) [^18^F]FDG NPV

Meta-analysis was conducted only on peritoneal metastases, because available data were insufficient for the analysis of distant metastases. Per-lesion analysis of the peritoneal metastases was carried out in four studies including 82 ovarian cancer patients (294 regions and 40 lesions). The pooled sensitivities of [^68^Ga]Ga-FAPI-04 and [^18^F]FDG were 97% and 70%, respectively; the pooled specificities were 93% and 88%, respectively; the pooled PPVs were 99% and 96%, respectively; the pooled NPVs were 86% and 43%, respectively (Table 6; Fig. 3) [27–30]. Three studies visually assessed the Eisenkop score or the peritoneal cancer index (PCI) for abdominopelvic tumour burden quantification on [^68^Ga]Ga-FAPI-04 and [^18^F]FDG PET/CT [27, 29, 30]. In particular, Liu S. et al. calculated Eisenkop score for both radiopharmaceuticals: [^68^Ga]Ga-FAPI-04 peritoneal score was significantly higher than [^18^F]FDG (27 vs 16), indicating that [^68^Ga]Ga-FAPI-04 detected a greater tumour burden [27]. Chen et al. and Liu Y. et al. reported a median PCI_FAPI_ significantly higher than PCI_FDG_ (15 vs 11 and 6 vs 4, respectively) [29, 30]. Moreover, Chen et al. showed that the PCI_FAPI_ had a stronger correlation with intraoperative PCI than PCI_FDG_ (*r*: 0.982 vs 0.867) [29]. At semi-quantitative analysis of PET parameters, median SUV_max_ and TBR of [^68^Ga]Ga-FAPI-04 were significantly higher compared with [^18^F]FDG in peritoneal metastases (SUV_max_: 3.91 vs 3.08; TBR: 4.77 vs 1.55, respectively) [30]. Conversely, no statistically significant difference was found between [^68^Ga]Ga-FAPI-04 and [^18^F]FDG qualitative [29] and semi-quantitative parameters evaluated in distant non-peritoneal metastases [26, 27, 30].

**Table 6:** Data for per-lesion qualitative analysis of peritoneal metastases in ovarian cancer

	Sensitivity (95% CI)		Specificity (95% CI)		PPV (95% CI)		NPV (95% CI)	
	^68^Ga-FAPI	[^18^F]FDG	^68^Ga-FAPI	[^18^F]FDG	^68^Ga-FAPI	[^18^F]FDG	^68^Ga-FAPI	[^18^F]FDG
Liu S. et al. [27]	1.00 (0.86, 1.00)	0.50 (0.31, 0.69)	0.00 (0.00, 0.79)	-	0.96 (0.80, 0.99)	1.00 (0.76, 1.00)	-	0.00 (0.00, 0.24)
Xi et al. [28]	0.97 (0.85, 0.99)	0.88 (0.73, 0.95)	1.00 (0.61, 1.00)	0.83 (0.44, 0.97)	1.00 (0.90, 1.00)	0.97 (0.84, 0.99)	0.86 (0.49, 0.97)	0.56 (0.27, 0.81)
Chen et al. [29]	0.97 (0.91, 0.99)	0.76 (0.65, 0.84)	0.99 (0.95, 1.00)	0.98 (0.94, 1.00)	0.99 (0.93, 1.00)	0.97 (0.89, 0.99)	0.98 (0.94, 1.00)	0.86 (0.79, 0.91)
Liu Y. et al. [30]	0.90 (0.80, 0.95)	0.61 (0.48, 0.72)	0.93 (0.69, 0.99)	0.71 (0.45, 0.88)	0.98 (0.91, 1.00)	0.90 (0.77, 0.96)	0.68 (0.46, 0.85)	0.29 (0.17, 0.46)
**Pooled** **[I**^**2**^**, *p*-value]**	0.97 (0.94, 1.00) [32.21, 0.22]	0.70 (0.56, 0.85) [82.23, 0.00]	0.93 (0.78, 1.07) [73.42, 0.01]	0.88 (0.69, 1.06) [65.64, 0.05]	0.99 (0.97, 1.00) [0.00, 0.81]	0.96 (0.93, 0.99) [0.00, 0.52]	0.86 (0.66, 1.06) [76.72, 0.01]	0.43 (−0.08, 0.93) [98.60, 0.00]

## Discussion

This systematic review investigates the overall diagnostic performance of FAPI radiopharmaceuticals compared with [^18^F]FDG in gynaecological cancers, with a meta-analysis comparing the diagnostic performances of both radiopharmaceuticals in ovarian cancer. All included studies were conducted with different FAPI radiopharmaceuticals labelled with gallium-68 (mainly [^68^Ga]Ga-FAPI-04) [21–30]. Although direct comparative data between different quinoline-based FAPI radiopharmaceuticals (e.g., FAPI-04, FAPI-46) are limited, these radiopharmaceuticals appear comparable in tumour and background uptake and can be considered as a single class [31]. However, studies suggest that FAPI-46 generally outperforms earlier versions, such as FAPI-04. Future studies with these next-generation FAPIs could demonstrate their potential to achieve superior imaging performance compared to [^18^F]FDG, exceeding the imaging outcomes reported in our study [17]. Despite the promising diagnostic performance of FAPI radiopharmaceuticals, their clinical implementation faces significant challenges. The rigorous and costly path to regulatory approval might result in a limited number of radiotracers being submitted for approval. Additionally, logistical and economical aspects, including the availability and cost of FAPI ligands, must be thoroughly evaluated. These considerations are crucial for the widespread adoption of FAPI radiopharmaceuticals in clinical practice.

[^68^Ga]Ga-FAPI-04 uptake of primary tumour was high and diffuse, similarly to that of [^18^F]FDG, in two uterine cancer patients [22]. This uptake pattern can mimic ^68^Ga-FAPI physiological uptake in endometrium and myometrium due to FAP overexpression during the cyclic endometrial remodelling in the pre- and peri-menopausal phases [21, 22, 32]. Likewise, [^18^F]FDG physiological uptake can be challenging, due to an increased cell glucose metabolism in the ovulatory and menstrual phases [7, 8, 33]. At visual analysis, no difference was found between [^68^Ga]Ga-FAPI-04 and [^18^F]FDG in the detection of primary tumour in cervical cancer [24, 25]. At semi-quantitative analysis, tumour to background (uterus) ratio was lower in ^68^Ga-FAPI compared with [^18^F]FDG. These results suggest that [^68^Ga]Ga-FAPI-04 is not useful for primary tumour staging in uterine and cervical cancer, due to its physiological accumulation in myometrium, hindering the evaluation of local tumour extent and invasion [22, 24, 25]. Based on our meta-analysis, the pooled sensitivity and specificity of ^68^Ga-FAPI radiopharmaceuticals and [^18^F]FDG were similar in primary ovarian cancer, suggesting that ^68^Ga-FAPI radiopharmaceuticals do not significantly enhance the role of PET in evaluating the primary tumour.

In cervical cancer, current studies showed that ^68^Ga-FAPI detected more metastatic lymph nodes than [^18^F]FDG in four patients [22–24]. One possible reason is the lower physiological background activity of ^68^Ga-FAPI compared with [^18^F]FDG, resulting in higher lesion-to-background values. As reported by Lyu et al., the diagnostic specificity of [^68^Ga]Ga-FAPI-04 is significantly higher than that of [^18^F]FDG [25]. One possible explanation is that ^68^Ga-FAPI uptake in reactive lymph nodes is lower than [^18^F]FDG uptake. We believe that ^68^Ga-FAPI could be useful in distinguishing metastatic from reactive nodes, thus allowing an accurate nodal staging. In ovarian cancer, [^68^Ga]Ga-FAPI-04 showed higher sensitivity and PPV for lymph node metastases than [^18^F]FDG, possibly due to the higher TBR of ^68^Ga-FAPI, suggesting its added value for detecting metastatic nodes [26–30].

Based on the results of our meta-analysis, ^68^Ga-FAPI has higher pooled sensitivity and PPV compared with [^18^F]FDG in detecting peritoneal carcinomatosis, enabling the identification of a greater number and larger extent of peritoneal foci [26–30]. Indeed, the PCI_FAPI_ were higher than PCI_FDG_ and closer to intraoperative PCI [29, 30]. A possible explanation are the significantly higher TBR values of ^68^Ga-FAPI, due to its lower physiological abdominal activity compared with [^18^F]FDG. These findings suggest that ^68^Ga-FAPI provides a more accurate assessment of the abdominopelvic tumour burden, making ^68^Ga-FAPI PET a promising non-invasive tool to predict tumour resectability and to better select patients for optimal cytoreductive surgery (i.e., R0 or R1 resection). Conversely, no significant difference was found between ^68^Ga-FAPI and [^18^F]FDG in detecting distant non-peritoneal metastases [26, 27, 29, 30]. Interestingly, ^68^Ga-FAPI showed lower diagnostic sensitivity for uterine metastases compared with [^18^F]FDG (16.67% vs 83.33%), because of its higher physiological uptake in the uterus [28].

Different ^68^Ga-FAPI semi-quantitative parameters have been analysed. Based on our results, their diagnostic significance remains uncertain. Larger studies are needed to standardise quantification assessment and to understand their clinical utility, before incorporating them into clinical practice.

In three of 10 included studies, ^68^Ga-FAPI PET was performed using a PET/MR scanner [22, 25, 28]. Despite the paucity of data, it can be anticipated that ^68^Ga-FAPI PET/MR could be a valuable tool for resectability prediction of ovarian cancer, providing a more accurate tumour burden estimation than [^18^F]FDG PET/CT. From a clinical point of view, physicians aim to an “all-in-one” imaging study, with best performances for primary, nodal, and distant disease.

Sun et al. recently conducted a systematic review and meta-analysis, updated to December 2023, including eight studies that compared ^68^Ga-FAPI with [^18^F]FDG PET in gynaecological cancers [34]. Seven of the articles evaluated in their analysis were also included in our selection [23–29]. Unlike our study, the authors focused only on the sensitivity of FAPI and [^18^F]FDG PET in the detection of metastatic lesions. Moreover, they did not perform a sub-analysis of the studies by tumour type in the detection of nodal metastases. In the assessment of peritoneal carcinomatosis, they included a study that combined data from pleural and peritoneal metastases [26]. Finally, Sun et al. did not describe semiquantitative data [34].

Our study has strengths and limitations. This is a systematic review and meta-analysis on the overall diagnostic performance of ^68^Ga-FAPI radiopharmaceuticals compared with [^18^F]FDG in gynaecological cancers, in particular cervical, uterine and ovarian cancers, with distinct results for each tumour type as well as for primary, nodal, and distant disease. However, the study is subject to several limitations, namely the consultation of a restricted number of databases, the small number of included studies, their limited sample sizes, and methodological heterogeneity, all of which may introduce bias. Consequently, not all studies provided sufficient outcome data for inclusion in the meta-analysis, which was ultimately performed only for the ovarian cancer subgroup. Nonetheless, despite the heterogeneity in study design among the included papers, which may affect the reliability of the findings, this meta-analysis yielded valuable insights regarding ovarian cancer. These methodological constrains highlight the necessity for well-defined, large-scale prospective clinical trials, with standardised patient cohorts, to reinforce the evidence base and enhance the robustness of future research in gynaecological cancer research.

## Conclusion

^68^Ga-FAPI radiopharmaceuticals show great potential as tracers for staging and restaging gynaecological malignancies. In particular, ^68^Ga-FAPI has demonstrated advantages over [^18^F]FDG in detecting nodal involvement in cervical cancer and in detecting nodal and peritoneal metastases in ovarian cancer. Larger prospective studies are needed to confirm these potential benefits and determine whether ^68^Ga-FAPI can replace or complement [^18^F]FDG in clinical routine for these indications.

## Supplementary Information

Below is the link to the electronic supplementary material.

Supplementary file1 (PDF 144 KB)

Supplementary file2 (PDF 415 KB)

## Data Availability

All data generated or analysed during this study are included in this published article and its supplementary information files.
