# Supplementary material for: Head-to-head comparison of fibroblast activation protein inhibitors (FAPI) radiopharmaceuticals and [18F]FDG in gynaecological malignancies: systematic literature review and meta-analysis
Source: Eur J Nucl Med Mol Imaging. 2025 Apr 25;52(11):3975–89. doi: 10.1007/s00259-025-07277-0 (PMC12397109; doi:10.1007/s00259-025-07277-0)
Supplement: Supplementary file 1 — Supplementary file1 (PDF 144 KB) [file 259_2025_7277_MOESM1_ESM.pdf]

ONLINE RESOURCE 1

Article title

Head-to-head comparison of fibroblast activation protein inhibitors (FAPI) radiopharmaceuticals and [^{18}F]FDG in gynaecological malignancies: systematic literature review and meta-analysis

Journal

European Journal of Nuclear Medicine and Molecular Imaging

Authors

Anita Florit, MD, Elizabeth J. De Koster, MD, Serena Sassano, MD, Lejla Alic, PhD, Giusi Pisano, MD, Floris H.P. van Velden, PhD, Salvatore Annunziata, MD, PhD, Irina Primac, PhD, Maria Rosaria Ruggiero, PhD, Cristina Müller, PhD, Evis Sala, MD, PhD, Wolfgang P. Fendler, MD, PhD, Giovanni Scambia, MD, Lioe-Fee de Geus-Oei, MD, PhD, Anna Fagotti, MD, PhD, Vittoria Rufini, MD, Angela Collarino, MD, PhD

Corresponding author

Prof. Vittoria Rufini

UOC Medicina Nucleare, Fondazione Policlinico Universitario A. Gemelli-IRCCS, Largo A. Gemelli, 8, 00168 Rome, Italy

E-mail: vittoria.rufini@unicatt.it

Supplementary Fig. 1 PubMed search string

((Gynecol*[tiab] OR Gynaecol*[tiab] OR Female[tiab] OR Vulv*[tiab] OR Ovar*[tiab] OR Endometri*[tiab] OR Uter*[tiab] OR Tuba*[tiab]) AND (Cancer[tiab] OR Carcinoma[tiab] OR Tumo*[tiab] OR Neoplasm[tiab] OR Malignan*[tiab]) OR "Genital Neoplasms, Female"[mh])

AND

((FAPI[tiab] OR "Fibroblast activation protein inhibitor"[tiab] OR FAP[tiab]) AND (FDG[tiab] OR 18F-FDG[tiab] OR Fluorodeoxyglucose[tiab] OR Fluoro-2-Deoxy-D-glucose[tiab] OR "Fluorodeoxyglucose F18"[mh]) AND (PET[tiab] OR (Positron[tiab] AND emission[tiab] AND tomograph*[tiab]) OR "Positron-Emission Tomography"[mh] OR PET-CT[tiab] OR PET-MR*[tiab]))

Supplementary Fig. 2 Embase search string

((“Gynecol*”.ab,ti OR “Gynaecol*”.ab,ti OR “Female”.ab,ti OR “Vulv*”.ab,ti OR “Ovar*””.ab,ti OR “Endometri*”.ab,ti OR “Uter*”.ab,ti OR “Tuba*”.ab,ti) AND (“Cancer”.ab,ti OR “Carcinoma”.ab,ti OR “Tumo*”.ab,ti OR “Neoplasm”.ab,ti OR “Malignan*”.ab,ti) OR "Genital Neoplasms, Female"[mh])

AND

((FAPI[tiab] OR "Fibroblast activation protein inhibitor"[tiab] OR FAP[tiab]) AND (FDG[tiab] OR 18F-FDG[tiab] OR Fluorodeoxyglucose[tiab] OR Fluoro-2-Deoxy-D-glucose[tiab] OR "Fluorodeoxyglucose F18"[mh]) AND (PET[tiab] OR (Positron[tiab] AND emission[tiab] AND tomograph*[tiab]) OR "Positron-Emission Tomography"[mh] OR PET-CT[tiab] OR PET-MR*[tiab]))
