# Supplementary material for: Head-to-head comparison of fibroblast activation protein inhibitors (FAPI) radiopharmaceuticals and [18F]FDG in gynaecological malignancies: systematic literature review and meta-analysis
Source: Eur J Nucl Med Mol Imaging. 2025 Apr 25;52(11):3975–89. doi: 10.1007/s00259-025-07277-0 (PMC12397109; doi:10.1007/s00259-025-07277-0)
Supplement: Supplementary file 2 — Supplementary file2 (PDF 415 KB) [file 259_2025_7277_MOESM2_ESM.pdf]

ONLINE RESOURCE 2

Article title

Head-to-head comparison of fibroblast activation protein inhibitors (FAPI) radiopharmaceuticals and [^{18}F]FDG in gynaecological malignancies: systematic literature review and meta-analysis

Journal

European Journal of Nuclear Medicine and Molecular Imaging

Authors

Anita Florit, MD, Elizabeth J. De Koster, MD, Serena Sassano, MD, Lejla Alic, PhD, Giusi Pisano, MD, Floris H.P. van Velden, PhD, Salvatore Annunziata, MD, PhD, Irina Primac, PhD, Maria Rosaria Ruggiero, PhD, Cristina Müller, PhD, Evis Sala, MD, PhD, Wolfgang P. Fendler, MD, PhD, Giovanni Scambia, MD, Lioe-Fee de Geus-Oei, MD, PhD, Anna Fagotti, MD, PhD, Vittoria Rufini, MD, Angela Collarino, MD, PhD

Corresponding author

Prof. Vittoria Rufini

UOC Medicina Nucleare, Fondazione Policlinico Universitario A. Gemelli-IRCCS, Largo A. Gemelli, 8, 00168 Rome, Italy

E-mail: vittoria.rufini@unicatt.it

Supplementary Fig. 3 Per-lesion analysis of the primary tumor in ovarian cancer. (A) ^{68}Ga-FAPI sensitivity; (B) [^{18}F]FDG sensitivity; (C) ^{68}Ga-FAPI specificity; (D) [^{18}F]FDG specificity; (E) ^{68}Ga-FAPI positive predictive value (PPV); (B) [^{18}F]FDG PPV; (C) ^{68}Ga-FAPI negative predictive value (NPV); (D) [^{18}F]FDG NPV

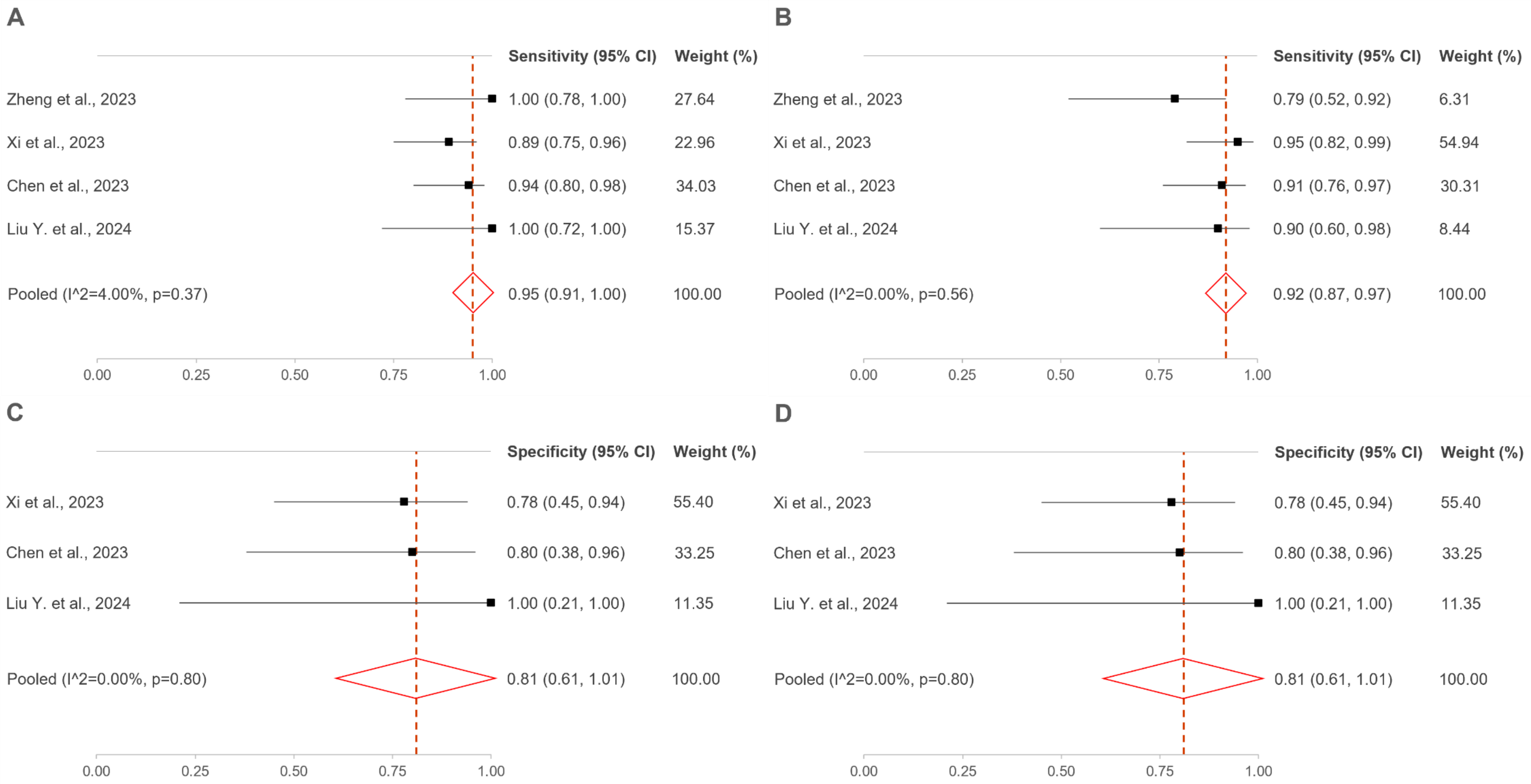

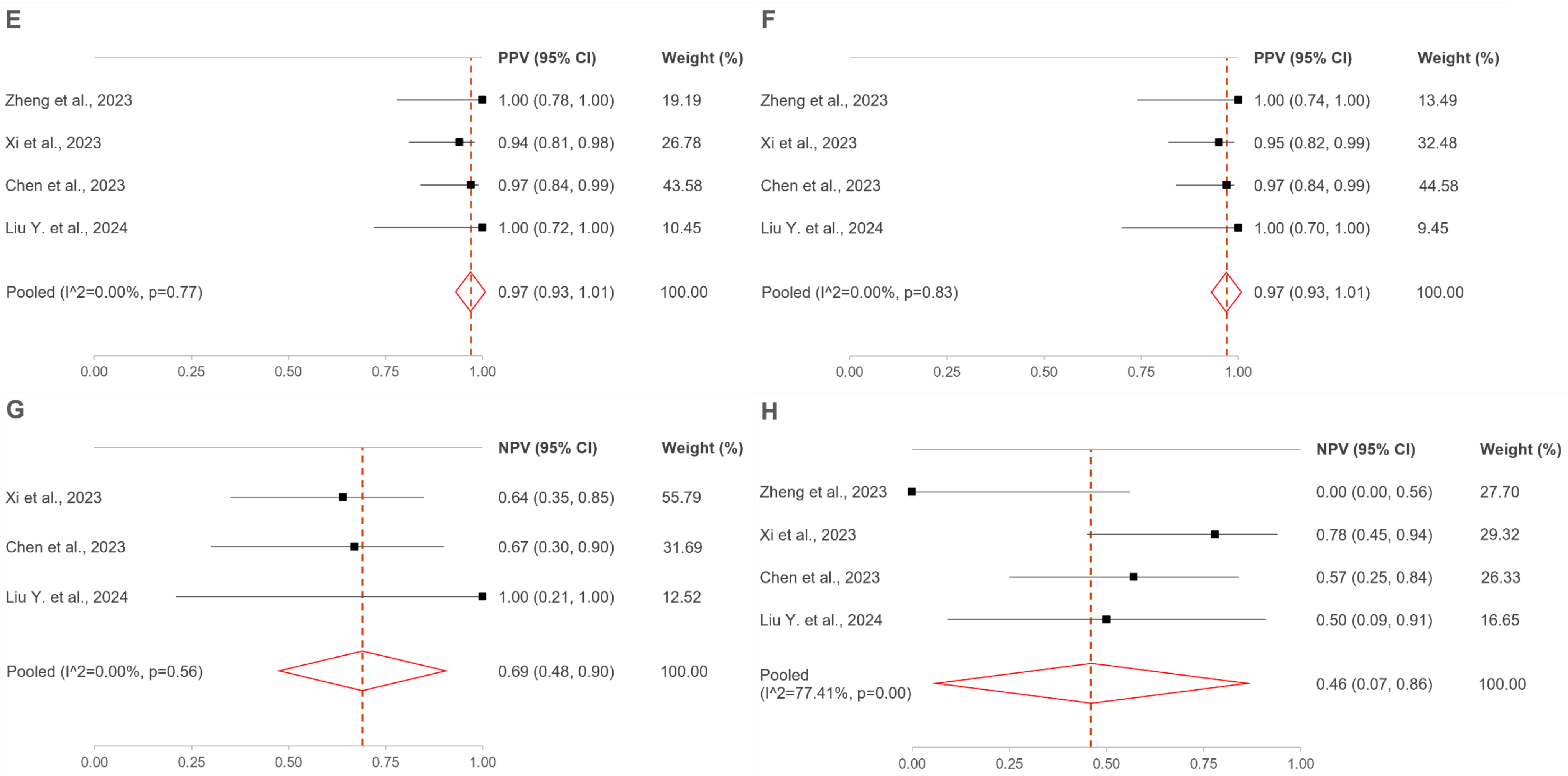

E
PPV (95% CI)
Weight (%)
Zheng et al., 2023
1.00 (0.78, 1.00)
19.19
Xi et al., 2023
0.94 (0.81, 0.98)
26.78
Chen et al., 2023
0.97 (0.84, 0.99)
43.58
Liu Y. et al., 2024
1.00 (0.72, 1.00)
10.45
Pooled (I^2=0.00%, p=0.77)
0.97 (0.93, 1.01)
100.00
0.00
0.25
0.50
0.75
1.00
F
PPV (95% CI)
Weight (%)
Zheng et al., 2023
1.00 (0.74, 1.00)
13.49
Xi et al., 2023
0.95 (0.82, 0.99)
32.48
Chen et al., 2023
0.97 (0.84, 0.99)
44.58
Liu Y. et al., 2024
1.00 (0.70, 1.00)
9.45
Pooled (I^2=0.00%, p=0.83)
0.97 (0.93, 1.01)
100.00
0.00
0.25
0.50
0.75
1.00
G
NPV (95% CI)
Weight (%)
Xi et al., 2023
0.64 (0.35, 0.85)
55.79
Chen et al., 2023
0.67 (0.30, 0.90)
31.69
Liu Y. et al., 2024
1.00 (0.21, 1.00)
12.52
Pooled (I^2=0.00%, p=0.56)
0.69 (0.48, 0.90)
100.00
0.00
0.25
0.50
0.75
1.00
H
NPV (95% CI)
Weight (%)
Zheng et al., 2023
0.00 (0.00, 0.56)
27.70
Xi et al., 2023
0.78 (0.45, 0.94)
29.32
Chen et al., 2023
0.57 (0.25, 0.84)
26.33
Liu Y. et al., 2024
0.50 (0.09, 0.91)
16.65
Pooled
(I^2=77.41%, p=0.00)
0.46 (0.07, 0.86)
100.00
0.00
0.25
0.50
0.75
1.00
